# *Tírala Plena*: findings from the formative research to inform the initiative “Reaching those most left behind through comprehensive sexuality education for out-of-school young people” in Colombia

**DOI:** 10.1080/26410397.2023.2267202

**Published:** 2023-11-20

**Authors:** Jair Vega Casanova, Johanna Blanco, Natalia Buitrago Rovira, Diana Matilde Pulido Jaramillo, Karen Adrians Pacheco, Alma Virginia Camacho-Hubner

**Affiliations:** a Professor, Department of Communication and researcher, PBX Research Group on Communication, Culture and Social Change, Universidad del Norte, Barranquilla, Colombia; b Advisor on Adolescence and Youth, UNFPA, Bogotá, Colombia; c Research Assistant, PBX Research Group on Communication, Culture and Social Change, Universidad del Norte, Barranquilla, Colombia; d Consultant, Development Curriculum “Tírala Plena”, UNFPA, Bogotá, Colombia; e Regional Technical Adviser, Sexual and Reproductive Health, Regional Office for Latin America and the Caribbean, UNFPA, Panama, Panama

**Keywords:** comprehensive sexuality education (CSE), adolescent pregnancy, prevention, STIs, HIV, GBV, parental practices, young migrants

## Abstract

This paper presents the results of formative research conducted from January to June 2020 in the Department of Atlántico, Caribbean region of Colombia, whose findings were used as inputs to design the national strategy for comprehensive sexuality education (CSE) outside school – *Tírala Plena* – including its curriculum. This is within the framework of the multi-country project coordinated by UNFPA and WHO aimed at generating evidence on the role of facilitators in the delivery of CSE in non-school contexts. The research was carried out in four municipalities in northern Colombia, in rural and marginal urban contexts with conditions of vulnerability for the adolescent population, including a strong presence of migrant populations from Venezuela. A total of 150 male and female adolescents ages 10–17 participated in the formative research. Workshops such as patchwork quilt, body mapping and talking maps were used as methods to gather information. The groups were divided by sex and age (10–13 years old and 14–17 years old). Knowledge, attitudes and social norms regarding adolescent pregnancy, sexually transmitted infections, including human immunodeficiency virus, and gender-based violence, were identified in adolescents (schooled and not schooled, but with minimal or no access to CSE). All of the above enabled us to establish a set of recommendations for the strengthening of the CSE strategy *Tírala plena*.

## Résumé

Cet article présente les résultats d’une recherche formative menée de janvier à juin 2020 dans le département d’Atlántico, région caribéenne de la Colombie, dont les résultats ont été utilisés pour concevoir la stratégie nationale d’éducation complète à la sexualité (ECS) en dehors de l’école, « Tírala Plena », y compris son programme d’études. Cela s’inscrit dans le cadre du projet plurinational coordonné par le FNUAP et l’OMS visant à produire des données sur le rôle des facilitateurs dans l’ECS prodiguée en milieu non scolaire. La recherche a été réalisée dans quatre municipalités du Nord de la Colombie, dans des contextes ruraux et urbains marginalisés avec des conditions de vulnérabilité des adolescents, notamment une forte présente de populations migrantes originaires du Venezuela. Au total, 150 adolescents des deux sexes, âgés de 10 à 17 ans, ont participé à la recherche formative. Des ateliers d’activités comme le patchwork, la cartographie corporelle et la cartographie sociale ont été utilisés comme méthodes de recueil des informations. Les groupes étaient divisés par sexe et âge (de 10 à 13 ans et de 14 à 17 ans). Des connaissances, des attitudes et des normes sociales concernant la grossesse des adolescentes, les infections sexuellement transmissibles, y compris le virus de l’immunodéficience humaine, et la violence sexiste, ont été identifiées chez les adolescents (scolarisés et non scolarisés, mais avec un accès minime ou inexistant à l’ECS). Tout ce qui précède nous a permis d’établir un ensemble de recommandations pour le renforcement de la stratégie d’ECS Tírala Plena.

## Resumen

Este artículo presenta los resultados de una investigación formativa realizada entre enero y junio de 2020 en el departamento del Atlántico, en la región caribe de Colombia, cuyos hallazgos se utilizaron como aportes para formular la estrategia nacional de educación integral en sexualidad (EIS) fuera de la escuela, “Tírala Plena”, así como su currículo. Esto ocurrió dentro del marco del proyecto multinacional coordinado por UNFPA y la OMS, cuyo objetivo es generar evidencia sobre la función de facilitadores en impartir EIS en contextos no escolares. La investigación se llevó a cabo en cuatro municipalidades del norte de Colombia, en zonas rurales y urbanas marginadas con condiciones de vulnerabilidad para la población adolescente, que incluye una importante presencia de poblaciones migrantes de Venezuela. En la investigación formativa participó un total de 150 hombres y mujeres adolescentes entre 10 y 17 años. Para reunir información, se utilizaron talleres tales como colcha de retazos, mapeo corporal y mapas hablantes. Los grupos fueron divididos por sexo y edad (de 10 a 13 años y de 14 a 17 años). Se identificaron los conocimientos, las actitudes y las normas sociales relativos al embarazo en la adolescencia, infecciones de transmisión sexual, incluido el virus de inmunodeficiencia humana, y violencia de género, entre adolescentes (escolarizados y no escolarizados, pero con mínimo o ningún acceso a EIS). Todo lo anterior nos permitió establecer un conjunto de recomendaciones para fortalecer la estrategia de EIS “Tírala plena”.

## Introduction

Sexuality is a human dimension that is culturally avoided in families, schools and the community, and as a result, a narrative is maintained that is based on the risk of pregnancy or STIs and the fear of violating social norms about unions, pleasure, power relations and reproduction.^1^ People have less evidence-based information and greater acceptance of myths and beliefs without evidence-based support, which is transmitted inter-generationally or among peers.^2^ It is, therefore, necessary to strengthen comprehensive sexuality education (CSE) in and out of school ^3–5^ with a positive approach in which sexuality is recognised as a human dimension that includes biological, cultural and social elements, where the enjoyment of human rights, including pleasure, free expression, autonomy and decision- making that contribute to well-being are more relevant than fear, silence and coercion.^6^

To support the dissemination and uptake of the International Technical and Programmatic Guidance on Out-of-School CSE,^4^ UNFPA Headquarters launched a three-year initiative entitled *Reaching those most left behind through CSE for out of school young people* in four countries (Colombia, Ethiopia, Ghana and Malawi).* Each of these four countries has selected a region to work with a particular group of adolescents and young people; the guidelines were used to design a plan tailored to their specific context, needs and life trajectories.

Alongside dissemination and implementation of the guidance, a key objective of the initiative is to build the evidence base on designing and delivering CSE in out-of-school contexts and for specific groups of young people: in Colombia, these are young people in communities with a large population of Venezuelan migrants, as the conditions of migrants and humanitarian contexts have presented specific challenges and barriers to CSE delivery. Aiming to design a comprehensive sexuality education (CSE) strategy – *Tírala Plena* – a formative research was conducted to determine the scope of CSE for boys, girls and adolescents between 10 and 17 years of age in the Department of Atlántico, Caribbean region of Colombia. The formative research was conducted between the months of January and May 2020, with the participation of a total of 150 boys, girls and adolescents. The results obtained from this research, together with a literature review – among others – were used to design the curriculum of *Tírala Plena*, the CSE strategy that has been implemented since 2021 in the Atlántico department.

For the purposes of the *Tírala Plena* and the formative evaluation research, four municipalities were selected in the Department of Atlántico, in northern Colombia: Barranquilla and Soledad (with higher population density and more urban cultural features), and Santa Lucía and Campo de la Cruz (more rural and less density populated). The two main characteristics of the municipalities and the selected population were: (1) the presence of a young migrant population from Venezuela, and (2) in contexts of vulnerability, their indicators concerning SRH are more precarious than the national consolidated figures across all groups in these contexts.

Historically there has been a relationship between the municipalities of the Department of Atlántico and Venezuela. In times of economic abundance in the neighbouring country, many Colombian families migrated to Venezuela to improve their living conditions, but the change in conditions forced the return and migration of family groups and people who were born or had lived in Venezuela for long periods of time, without any roots in the ways of life of the territories in the Department of Atlántico.^7^

For 2020, the Venezuelan migrant population in these municipalities was already significant: Barranquilla (7.66%), Soledad (4.73%), Campo de la Cruz (7.08%) and Santa Lucía (10.23%).^7^ Although specific data on the number of adolescents in each municipality are not available, it is known that in 2019, almost 4 out of 10 Venezuelan migrants based in Colombia were children and adolescents.^8^ In most cases, adolescent migrants from Venezuela live in the same contexts as adolescents from the receiving communities, facing similar circumstances of vulnerability, such as precarious economic income, little or no access to activities that allow them to generate life projects, and no access to CSE.

Regarding indicators concerning SRH, the *Tírala Plena* project, based on national sexual and reproductive health and rights (SRHR) priorities and evidence of the potential of CSE to generate transformations in these areas, prioritised the prevention of adolescent pregnancy,^9^ and reduction in gender-based violence (GBV)^10^ and sexually transmitted infections (STIs), including Human Immunodeficiency Virus (HIV),^9^ in the adolescent population aged 10–17 years.

With regard to CSE, Colombia has dedicated decades to the promotion and guarantee of SRHR, with two main programmes: in the educational sector, the Education for Sexuality and Construction of Citizenship Program (PESCC),^11^ which relies on current national regulations, such as the General Law of Education (1994), and provides guidelines for the construction of pedagogical projects in schools; and in the health sector, Youth-friendly Health Services for Adolescents and Youths (SSAAJ),^12^ a process in which health institutions create spaces and forms of comprehensive and differentiated care for the population between 10 and 29 years of age.

Colombia has made slow progress in coverage (2019) in basic secondary education (72.06%) and vocational secondary education (44.55%).^13^ State guidelines have been issued to incorporate children and adolescents from Venezuela into the education system (Circular 016 of 2018).^14^ However, although most children and adolescents are in school, in most cases – especially in rural areas and marginal urban contexts – they are not receiving quality CSE, largely due to the low coverage and fidelity of implementation of the programmes.^11^ For this reason, programmes such as *En la Jugada*, which has a national scale and is implemented by the Colombian Institute of Family Welfare (ICBF) in non-school contexts (institutional and community spaces), are of great importance. This ICBF programme was the basis for developing the *Tírala Plena* project in the Department of Atlántico, targeting adolescents from the most vulnerable sectors of the population, both in and out of school, between 14 and 28 years old, including migrant adolescents from Venezuela.

### Objective

The main objective of this paper is to present the findings from the formative research whose purpose was to identify what knowledge, attitudes and social norms adolescent girls and boys aged 10–17 in the selected municipalities have in relation to sexuality, teen pregnancy, STIs including HIV, and GBV.

## Methods

### Literature review

A narrative bibliographical review of published articles and documents was carried out in order to search for key words. The terms included in the review were: “teenage pregnancy”, “Sexually Transmitted Infections”, “HIV”, “AIDS”, “Gender-based violence”, “Barranquilla”, “Campo de la Cruz”, “Santa Lucía”, “Atlántico”, “Soledad”, “boys”, “girls”, “adolescents”, “comprehensive sexuality”, “migration”, “Venezuelan migrant population”, “sexual abuse”, “LGTBI population”, “teenage pregnancy in Colombia”, “STIs in Colombia”.

The selection of documents was based on criteria such as bibliographical reviews, indexed journals, documents from official state institutions, journalists’ reports and/or news events, and those that included some type of quantitative research, from 2010 onwards. A total of 45 bibliographical sources were consulted, of which 21 were broadly included within the writing of the literature review.

### Data collection

For data collection from adolescents, memory workshops (“patchwork quilts”) ^15^ and talking maps workshops were developed. The memory workshops (conducted by the research team of Universidad del Norte) are an interactive ethnographic method, which appeals to everyday memories, rather than opinions, that are captured in individual drawings that are then narrated (i.e. assembled into a “patchwork quilt”), allowing for collective analysis. The two patchwork quilts that were made in Santa Lucia and Soledad were based on indications such as: draw a moment in which you remember an act of GBV occurring. The workshops were complemented with a questionnaire with flexible questions and open answers, based on the topics included in the research questions. These questions focused on knowledge (What knowledge do you have?), attitudes (How much do you agree? and perceived social norms (What is acceptable to the majority?) about adolescent pregnancy, GBV and STIs, including HIV. Questions about previous access to CSE and the quality of the training received were also included.

Two interactive talking maps workshops were held, a technique that consisted of the participants locating the most frequented places in their municipality on a map and pasting photographs of these places in their correct locations. The participants are asked a series of questions that allow them to establish the characteristics of these places. The technique was satisfactorily applied in mixed groups of 8 women and 5 men between 14 and 17 years of age in two of the four municipalities. In total, in the four municipalities, 16 workshops were conducted with adolescents, separated into men and women and also by age (10-13 years old and 14–17 years old). These talking maps made it possible to identify the presence and power relations in terms of GBV, teenage pregnancy and STIs that shape the space inhabited by young people. Table 1 consolidates the characteristics of the adolescents participating in the research.

### Data analysis

For the analysis, we used the grounded theory perspective of Strauss and Corbin,^16^ initially through collective discussion among members of the research group in each of the workshops. This allowed us to carry out an open coding to progressively identify the categories that were becoming saturated and the information gaps. It also allowed us to refine the methodology for subsequent applications of the data collection instruments. The procedures were improved and new questions were added. This process included listening to the recordings, reading the reports, and discussing the experiences of the moderators and rapporteurs of each focus group or interview.

At a later stage, based on a microanalysis, coding was carried out by establishing the meanings resulting from the analysis of entire paragraphs, situations or complete experiences, or the narrative of a participant during a workshop. This exercise culminated in a set of organised data, based on the initial categories of the study, with which the final report document was prepared.

### Ethical considerations

The study was approved by the Research Ethics Committee of the Universidad del Norte, confirmed by appraisal report No. 213 dated July 30, 2020. Participants signed a consent form prior to filling out the questionnaires, with their authorisation to participate voluntarily. In the case of minors (10–17 years old), the parents signed an assent, at the time of the convocation, when they authorised their children’s participation in the workshops, after explaining to them the purpose of the study. Subsequently, the minors also signed a consent form expressing their voluntary participation, prior to the start of the workshops.

## Results

### Literature review

In three of the four selected municipalities, the fertility rate in 2018 for girls aged 10–14 years was higher than the country average of 2.83 per 1,000 girls: Barranquilla (3.02), Campo de la Cruz (4.57) and Santa Lucia (5.23), while in Soledad it was lower at 1.66 girls per 1,000.^17^ Likewise, the fertility rate for adolescents aged 15–19 years was higher than the national average (61.11 per 1,000 adolescents): Barranquilla (84.44), Campo de la Cruz (119.85) and Santa Lucía (115.45), but lower in Soledad (52.47).^17^.

There is no specific information on Venezuelan migrant adolescents in relation to adolescent pregnancy in these municipalities. However, in 2019, teenage pregnancy among Venezuelan migrant minors between 10 and 17 years of age (8.9%) was more than double that of Colombian minors (3.5%).^8^ This population encounters barriers in access to health care, especially to family planning and sexual and reproductive health services.^18^

With respect to STIs/HIV, in 2019, in Barranquilla the number of HIV cases increased to 547 and the incidence rate to 44.2 per 100,000 inhabitants, the second highest in the country^19^; in Santa Lucia there were five cases of HIV, two cases of congenital syphilis and nine cases of gestational syphilis^20^; in Campo de la Cruz, six cases of gestational syphilis and five cases of HIV;^21^ and in Soledad, 340 cases of pregnant women from Venezuela with HIV and gestational syphilis in 2018.^22^ Immigrant adolescents in most cases do not have easy access to a health institution, which does not allow them to have an early diagnosis and timely treatment.^23^

Regarding GBV indicators, between 2016 and 2019, 1076 medical-legal examinations for alleged sexual offenses of Colombian and Venezuelan nationals in minors between 12 and 17 years old, were registered in Barranquilla.^24^ For the same period, in Soledad, 11 cases were registered in minors between 6 and 17 years old.

In 2019, sexual crimes were up to 80% more frequent among women aged 6–17 than older women in Barranquilla and Soledad. The presumed aggressor was a relative in 48.86% (Barranquilla) and 42.85% (Soledad) of the cases. Cases of GBV are undoubtedly higher than recorded because of barriers to reporting cases and a lack of attention to the immigrant population.^24^ Due to economic dependence on their partners, little or no family support network, and barriers to access to health, protection and work, migrant Venezuelan women are more vulnerable to GBV.^24^

Regarding knowledge, attitudes and social norms in relation to sexuality, adolescent pregnancy and gender-based violence, no specific data were found on the adolescent population in the selected municipalities and no updated data at the national level. The most recent data are from the 2015 National Demographic and Health Survey.^25^

According to this survey, female adolescents aged 13–14 and 15–19 reported significant knowledge of the male condom (94.6% and 98.4%), the pill (86.1% and 96.2%), the injection (79% and 95%) and female sterilisation (74.7% and 88.3%). The figures for male adolescents of the same ages were: of the male condom (95.5% and 99%), the pill (73.2% and 90.3%), injection (56.4% and 80.7%) and female sterilisation (66.6% and 83.2%). The same survey found that at least six out of 10 women and five out of 10 men under 20 years of age were aware of some or all of the cases in which abortion is decriminalised in Colombia.^25^

Likewise, by 2015, approximately four out of 10 women and men aged 13–19 years knew one or more symptoms of an STI in a man or a woman. Likewise, 97.2% of women and 96.4% of men aged 13–24 years had heard about HIV/AIDS and seven out of 10 women and men of the same age reported knowledge about their forms of protection. The comprehensive knowledge about HIV/AIDS in women and men aged 13–17 years for the same date were at 25.1% and 23.1%, respectively.^25^

Regarding gender-based violence, in 2015, at the national level, 2.8% of men aged 10–13 years and 2.5% of men aged 13–19 years agreed that sometimes it is okay for men to hit their partners. Likewise, 10.3% and 12% respectively agreed that it is justified to hit a partner when she has been unfaithful.^25^

It was found that the programmes and content of most of the interventions on CSE and adolescent SRHR provided in the Atlántico department – such as the Crisálida program at the departmental level, the SSAAJ program (Santa Lucia), the Sex Education and Citizenship Construction Program (PESCC) (Barranquilla) and the Inter-institutional Advisory Committee for the Prevention of Sexual Violence and Comprehensive Care for Children and Adolescents Victims of Sexual Abuse (Soledad) – correspond to public policies.

The programmes that have had greater coverage at the national level, at least in terms of public policy orientation, including the municipalities in this study, are the PESCC and the SSAAJ. However, in the first case, the evaluation conducted on the PESCC concludes that despite the relevance and quality of the programme it has had low coverage, implementation and involvement, greatly affected by the fidelity of its implementation.^11^ Likewise, the evaluation of the SSAAJ showed that despite the quality and relevance of the model, there are limitations in its implementation and cultural barriers to its use and that greater coordination with youth organisations is required, as well as spaces that involve adolescents who have not started their sexually active life.^12^

Finally, some publications from 20 years ago were found related to research on adolescence and sexuality in municipalities in the area where the formative research was conducted. They show, for example, how there are restricted scenarios for talking about sexuality within the family, since only moral judgments are expressed there, and in educational institutions only sporadic talks are held, basically on reproductive health.^26^ Likewise, it was found that sexuality is a topic that should not be talked about, generally associated with sexual intercourse where men are the ones who assume an active role and gender violence is significantly legitimised.^27^

Other studies about CSE in nearby municipalities, offered evidence that strategies based on dialogue and debate contributed to the adolescent population being able to talk openly about sexuality and to establishing better communication with teachers and health system officials.^28^ The adolescent population were able to identify gender-based violence and sexual violence in their daily lives, as well as to question social imaginaries that legitimise them, and to talk openly about them.^29^

### Formative research

The results of the formative research are presented below in accordance with the main objective. The findings are organised by topics and within each topic by knowledge, attitudes, and social norms, including in some cases, differences by sex, age or geographical location. The differences in the adolescent migrants from Venezuela who participated in the research are also indicated.

For purposes of describing specific “quantities”, throughout this section we will use the word “most” to refer to when more than half of the participants agreed on a response; “few” when the percentage was less than half; and “some” when 1, 2 or 3 participants had the same response or perception.

**Table 1.** Adolescent participants in the research

Municipality	Women		Men	
	10–13 years old	14–17 years old	10–13 years old	14–17 years old
Santa Lucía	12	12	12	12
Campo de la Cruz	12	10	13	11
Soledad	15	9	12	0
Barranquilla	3	6	4	5
Total	43	37	41	29

Participants were recruited through institutions that work with adolescents in non-school contexts. Some participants were schooled; however, questions were included in the focus groups that showed that they had not received CSE.

### Sexuality and adolescent pregnancy

#### Sexuality

The proven *knowledge* in most of adolescents (10–17 years old) in the study, male and female, shows an association of sexuality with sexual activities like “having sex”, assumed as genital (moreover penetrative) and heterosexual sexual practices. When adolescents talk about sexuality, they enunciate it from a position assumed as active by men and passive by women, understanding that men are the ones who propose to have sex or who perform the action “I did to … ”, while women are the ones who receive the action “he did to me, he had me, he hurt me”. When referring to sexuality, a few women include other dimensions indicating that it goes beyond genitality such as respect for the body and the construction of affective relationships.

Moderator: *What does come to your mind when you hear the word “sexuality”?*

Respondent: *Sex. (giggles)*.

Moderator: *ah?*

Respondent: *Sex.*

Moderator: *Ajá … what else?*

Respondent: *Pregnancy (in a very low voice tone)*.

Moderator: *ah? I didn't listen properly … did you just say sex?, what is that?*

Respondent: *Sexual relations (Laughing)*. (Workshop, female adolescents, 10–13 years old, Barranquilla).

Only female adolescents between 14–17 years of age have any information on SRH, specifically related to vaginal cytology. In Santa Lucía, they do not know what it consists of, while in Barranquilla they know what it is and declare that it is used to detect STIs.

Regarding *attitudes towards sexuality*, in adolescents between 10 and 13 and females in the more rural municipalities (Santa Lucía and Campo de la Cruz) there is a more open attitude and greater interest towards dialogue on self-care, self-esteem and pregnancy at an early age. They consider their mothers to be a reliable source for talking about sexuality, especially the prevention of sexual violence. In the urban context, on the contrary, adolescents in this same age and gender group consider that sexuality remains a taboo for their guardians.

Regarding social norms, in general, talking about sexuality (in both age groups) is still perceived as something uncomfortable, generating embarrassment or shame, especially when it is with adults or strangers. This attitude is also manifested with gestures and laughter during workshops.

#### Adolescent pregnancy

Talking about pregnancy**,** adolescents’ *knowledge* is mostly the understanding that pregnancy has to do with sexual intercourse, but in some cases, there are some inaccuracies in the information they have. Few male adolescents or girls aged 10–13 associate pregnancy with sexual intercourse. Some adolescent girls aged 14–17 in Santa Lucia do not know their menstrual cycle and they do not relate it to pregnancy. Also, there are adolescent men aged 14–17 in Barranquilla who assume that they can identify the presence of symptoms in women just one week after becoming pregnant.

Adolescents regarded *adolescent pregnancy* negatively, as something unfortunate that would hinder their life plans and cause problems with their family. This attitude is based on what they observed with their peers who have had this experience and, in many cases, have abandoned their studies to assume the “responsibilities” that society traditionally assigns. To women, it is the care of their children and the home; and to men it is working to support the new family, perpetuating traditional gender roles.

Moderator: *And do any of you want to be a mother? … look how she makes her face (giggles)*

Respondent: *I want to be a mother, but when I have my studies, my goals, and I have my own home* … 

Moderator: *And who have you heard talking about this?*

Respondent: *My mother.*

Moderator: *What has your mother told you?*

Respondent: *She tells me that if I let my boyfriend “eat” me, I'll get pregnant and that hurts. And she tells me: if you get pregnant, you just leave.* (Workshop, female adolescents, 10–14 years old, Santa Lucía)

Regarding *social norms*, specifically about social and family sanctions against pregnancy, adolescents disagree with the hostile way that parental figures normally act or react towards them when they ask about pregnancy prevention. They feel that instead of advising and guiding, parents scold and threaten to “kick them out”. On the other hand, in some circumstances there is a social acceptance of pregnancy in adolescence when it occurs within a relationship that is considered “serious”. It is simultaneously normalised that “the husband takes her to his house” and “he starts working while she assumes the household chores” and this may encourage premature unions with high risks.

There is a positive conception of maternity, when considering the child as a “blessing from God”. In general, however, motherhood is only accepted when other goals are achieved, even though their referents of femininity are associated with motherhood with numerous children and status of marriage. Female adolescents aged between 10 and 13 years old mention that they are too young to have boyfriends.

About *modern contraceptive methods*, there is very superficial knowledge, unspecific or misinformation among the general target population. The most mentioned way of prevention is condom. In rural areas, adolescents between the ages of 10 and 13 know about condoms but they have never seen them or know how to use them, although they say they have received training in this regard.

The adolescent population from 14 to 17 years old, in Barranquilla, an urban area, has more information on how to use a condom, including checking the expiration date, not keeping it in their wallets, and being careful when handling it. Some adolescents from rural areas, between 14 and 17 years of age, mention condoms, but not much knowledge about its use is evident.

Moderator: *How can you protect yourself from a pregnancy or STI?*

Respondent: *With condom, with injections, pills, and device* … 

Moderator: *To prevent a sexually transmitted disease you have to be sure about condom expiration date and do not keep it in your wallet. If you keep it in your wallet, it won’t work, be careful with the expiration date, if it breaks, then it doesn't work anymore. You have to keep it in the box where it comes from and do not play with that … That comes with a liquid-ish, you can't be playing with that.* (Workshop, female adolescents, 14–17 years old, Barranquilla)

In general, adolescent women of both age ranges and from rural and urban areas, identify the pills and subdermal and intrauterine devices as contraceptive methods, but do not know how they work. Finally, adolescent women from 14 to 17 years old in urban areas know the “morning after pill” but they consider it as a contraceptive method. It was evident that there is no knowledge about access to contraceptive methods through the health system.

No negative attitude towards the use of condom is expressed in the target population. In some cases, adolescents from rural area between 10 and 13 years old, assume that contraceptives cause weight gain, and that is why a woman who is using them is easily identifiable.

Towards the use of the condoms and prevention, as *social norms*, in Barranquilla, some adolescents between the ages of 14 and 17 consider that although it is not well regarded that a woman requests the use of a condom from her partner because she is judged as “promiscuous”, it is time to change that concept because women have to take the initiative to protect themselves.

In general, there is no clear knowledge about *voluntary interruption of pregnancy (VIP)* as a right or as an option in a situation of unwanted pregnancy, or at an early age.^†^ Adolescents from all ages express a negative attitude towards VIP, even though abortion has been legal in Colombia since 2006***.*** Adolescents from 10 to 13 years old, in Soledad (urban area) consider that grandmothers and mothers are the ones who do not agree with the interruption of pregnancy. On the other hand, adolescents between the ages of 14 and 17 consider the only exception would be when it is a result of sexual assault. There is a punitive social norm about abortion since it is considered that “the baby is not to blame” for the decisions of adolescents/adults.

### STIs including HIV

In most adolescents there is confusion and little to no information / *Knowledge* regarding the identification, transmission, and prevention of STIs, including HIV. Female adolescents confuse HIV with human papilloma virus (HPV), even those who have already had the HPV vaccine. Adolescents from 14 to 17 years of age in Santa Lucía are clear about the difference between HIV and AIDS, but not adolescents of the same age range in Barranquilla, who confuse the infection with the disease.

Moderator: *HIV is the same than AIDS?*

Respondent 1: *It is the same.*

Respondent 2: *Yes, it's the same thing.* (Workshop, male adolescents, 14–17 years old, Barranquilla)

Regarding STIs and HIV transmission, female adolescents aged between 10 and 13 in rural areas declare that they know about the modes of transmission of STIs, including HIV, but they have incorrect information. Adolescents from 14 to 17 years of age from urban areas have greater knowledge about the modes of transmission of STIs and HIV. The latter population identifies sexual intercourse, injections, and vertical mother–child transmission as modes of HIV transmission, but they still hold beliefs such as transmission by kissing.

Although some adolescents declare that condoms are necessary for prevention, participants have not necessarily had contact with them, nor do they know how to use them, although they say they have received training in this regard. Female adolescents from 10 to 13 years of age in Santa Lucia mention condoms as a way of preventing HIV; however, they also consider that it is prevented with a vaccine, probably due to confusion with HPV.

Female adolescents from rural areas, in both age ranges, do not know about vaginal cytology (pap smear). Contrary to female adolescents between 14 and 17 years of age in Barranquilla (urban area) who not only know about vaginal cytology and its advantages for prevention of STIs, but they also show a positive attitude towards performing a vaginal cytology to detect STIs. Additionally, in these urban areas there are less negative attitudes in relation to sexual diversity.

Adolescents from rural areas associate HIV with “faggots”, “gays” or lesbians, referring to homosexual people, expressing rejection and mistrust because considering them transmitters of the virus or “of AIDS”. Discriminatory *attitudes* are included, such as “not breathing” when a homosexual person who is “supposed to have AIDS” passes by. This does not happen in urban areas because there are less negative attitudes in relation to sexual diversity.

Female adolescents living in urban areas consider that *social norms*, such as judgemental attitudes towards women who use contraceptive methods or ask their partners to use condoms, persist, and assume that it is time to change them because women have to take the initiative to protect themselves.

### Gender-based violence

In general, male and female adolescents from urban and rural areas, have little to no *knowledge* about GBV. Although GBV is present in most environments of their daily lives, they do not identify it as such. There is confusion between GBV and domestic, street or criminal violence. After clarifying the concept, they identified various situations of GBV, including street harassment, abuse by peers, stalking, and sexual assault. Male adolescents expressed that woman should not be mistreated, but justified violence when women's behaviour violates their masculinity, such as when women are unfaithful.

Female adolescents declared experiencing GBV from the fear generated by walking alone through some places perceived as dangerous in their communities – streets, public squares, parks and playing fields. They identify the male figure as potentially dangerous, determining a permanently defensive *attitude*. Female adolescent migrants from Venezuela, aged 14–17 years, had difficulty recognising their own bodies, did not feel comfortable in body mapping activities, probably due to previous experiences of sexual violence.

Female urban adolescents, unlike their rural counterpart, express an attitude of rejection towards street harassment. However, this rejection occurs when the “compliments” are considered vulgar or do not come from men they “like”. Male adolescents consider that women who like those “compliments” are “horny” women, but not in the case of their girlfriends not their friends.

Moderator: *Have you ever blow a compliment like that? When a woman passes by with tight clothes or mini skirt … a compliment that you can say “wow, that´s a great compliment”*.

Respondent: *Well, well, my girlfriend likes that.*

Respondent: *You have to “blow compliments” and lots of that!*

Respondent: *Well, I blow compliments on her, but it is joking. With time, this looks awful.*

Respondent: *And I “compliment” my female friends but they like that.*

Moderator: *Women like men [to] blow compliments like that?*

Respondent: *Well, some of them, the ones that are horny.* (Workshop, male adolescents, 14–17 years old, Barranquilla)

In relation to *sexual abuse*, most men blame women for inciting it or families for not preventing it. Both sexes react more negatively to sexual abuse when it involves under-age girls.^[‡]^ However, although this is legally considered rape, there is a condescending attitude towards adolescent women under 14 years old pregnant by older men where there is a large age difference.

In fact, romantic relationships between adolescent girls and older men are normalised, even among adolescents aged 10–13. During workshops, adolescents told stories of adolescents in love with older men, justified because they are more “sincere” or “give them money”.

Many of the adolescents, male and female, have an *attitude* that justifies harassment and abuse. They indicated that this happens because some women “like it”, “incite” men with their clothes, “provoke men to hit them” or “do not earn their respect”. Some even point out, that a woman who was abused must “look like she was raped” or it is assumed that it did not happen.

Moderator: *Is it her fault? true or false?*

Respondent: *That´s true.*

Respondent: *She is the one that is provoking to be rape.*

Respondent: *I think that is true because she is the one that has to make herself respected.*

Respondent: *She has to wear the skirt to the knees.*

Respondent: *She is the one provoking, make herself seen to be raped.*

Moderator: *Well, then, when we as men wear shorts, someone could come and touch us just because we are wearing shorts?*

Respondent: *No.* (Workshop, male adolescents, 10–13 years old, Campo de la Cruz)

### Access to CSE

Although a few adolescents have obtained information on sexuality from institutions and individuals who could be appropriate sources, there was no reference to receiving an offer of quality CSE.

From the field diary, it was identified that for female adolescents aged 14–17 years in Barranquilla who are linked to the ICBF’s Substitute Homes programme, the right to health, including sexual and reproductive health, is among the guarantees of their rights. The rest of the target population do not appear to have received or have access to CSE. Mothers are considered as a reliable source to talk to about sexuality, especially the prevention of sexual violence; however, their advice can be interpreted more as warnings for prevention than guidance on how to live sexuality. In fact, in the urban context, on the contrary, adolescents aged 10–13 years consider that sexuality is still a taboo for their guardians.

### Care pathways for SHR and GBV

Male and female adolescents from all the municipalities and all age groups do not know the SRH care pathways. Only adolescents from 10 to 13 years of age in Santa Lucia identify the health services, because they saw an advertisement on a poster in the healthcare centre. In general, adolescents consider it essential to receive information about SRH, but they do not trust health services, including youth-friendly health services, because they believe that if they consult them or ask for condoms, they will be judged or laughed at. Adolescents from rural areas affirm that the hospital is in poor condition, which means that GBV cases are sent to Barranquilla. In case of pregnancy, because they are minors, they would go to the ICBF.

There is partial knowledge of the care pathways for GBV. In general, adolescents mention entities to go to report GBV cases, but without fully understanding the pathway. In fact, they do not conceive they have a right to access institutional attention or support in relation to either GBV or SRH. Adolescents do not trust reporting to the police, who they consider would not believe them, and in some circumstances, they would rather choose to go to the Family Police Station, a separate institution that deals with cases of domestic violence.

In addition to the lack of knowledge of where to go in case of a GBV complaint, men and women consider that there is no response to GBV, because neither at home, nor at school, nor in the institutions are they believed when they report any type of abuse. They distrust the few institutions they identify, such as the Family Police Station and the hospital. They think that their reports will not be taken seriously. In fact, most female adolescents consider institutions such as the police to be facilitators of this type of violence. Adolescents also do not trust their parental figures, particularly when the complaint falls on a relative.

Moderator: *What can your cousin do if something like this happens (sexual abuse)?*

Respondent: *To report.*

Moderator: *Where can she report?*

Respondent: *Police station.*

Respondent: *Sometimes you tell your mom that a relative id rapping you, abusing you, touching you … , and she won’t believe us.* (Workshop, female adolescents, 10–13 years old, Sant Lucía).

## Discussion

Although in some cases adolescents were identified as having obtained information on sexuality from institutions and other people who could be considered adequate sources, it cannot be said that this information is sufficient to satisfy all the concerns and needs of this population related to CSE, including SRHR. The formative research showed that the knowledge that the participating adolescent population has about sexuality, adolescent pregnancy, modern contraceptive methods, voluntary interruption of pregnancy, STIs including HIV, GBV and the SRH, and GBV care pathways for adolescents and young people is insufficient. It was evident that most of the young participants' knowledge is limited to the biological and genital aspects of sexuality. There is a lack of scientific foundations, and their thoughts are permeated by false beliefs, which in some cases, leads to a negative assessment of sexuality and everything related to this dimension of human life, even causing shyness and moral restrictions about speaking openly about what they know, think, or believe.

As in previous decades,^26–28^, there is still no evidence of an open attitude when talking about sexuality, much less when referring to their own experience. Generally, there is shame, as well as frequent laughter when talking about the subject. There is evidence of social norms that mean talking about sexuality is perceived as shameful.

Even though negative attitudes still persist towards talking openly about sexuality, or protective sexual behaviours such as using modern contraceptives methods, and stigmatising people who are HIV positive and people who belong to the LGBTI community, there is a more open attitude and more interest towards dialogue about self-care, self-esteem and pregnancy at early age.

Differences were found between groups of adolescents in rural and urban municipalities. In urban adolescents from both age groups, there is more information and fewer myths about sexuality, modern contraceptive methods and STIs, including HIV. In urban areas, such as Barranquilla, and in the 14–17-year-old age group, there is greater knowledge, more positive attitudes and social norms more in line with a sexuality understood from a rights rather than a moral perspective. There are no major differences between men's and women's knowledge of these topics. There is greater knowledge among the older adolescent population.

The adolescents in our study have a negative attitude towards adolescent pregnancy, as they consider it unfortunate for their life plans. Likewise, there is a positive attitude towards the use of condoms and only in some cases a negative attitude towards the use of contraceptive pills. However, there is still a great deal of misinformation about the association between the menstrual cycle and sexual relations with pregnancy and a superficial knowledge or lack of knowledge about modern contraceptive methods and voluntary interruption of pregnancy, which does not contribute to the prevention of adolescent pregnancies. This low level of knowledge about modern contraceptive methods and about abortion contrasts with the significant knowledge about these topics found in the adolescent population at the national level in the latest version of the ENDS (National Demographic and Health Survey).^25^

Social norms regarding adolescent pregnancy do not contribute to prevention either, as they are ambiguous: on the one hand, adolescent pregnancy is condemned by adolescents and their families, is considered a consequence of disobedience to the mandates and limits established within the family and reinforced by society, but once the child is born, it is assumed to be “God's blessing” and motherhood is assumed to be the central reference point for being a woman. Likewise, at the normative level, on the one hand, the adolescent girl is encouraged to form a couple with the father of her child to legitimise the pregnancy and, on the other hand, it is still seen as negative for a woman to take the initiative in the use of contraceptive and protective methods. All this leads to new pregnancies in adolescence. This could be related to the fact that the fertility rates of girls between 10 and 14 years old and between 15 and 19 years old are mostly higher than the national average.^17^

In the participating population, there is confusion or little information or mistaken beliefs about the identification of STIs, including HIV, as well as their prevention. Although in some cases they state that they have the information, it is not correct. They have some information about prevention methods such as condoms, but in most cases, they have not had contact with them. They also do not know about Pap smears. Adolescent girls aged 14–17 years in urban areas are more knowledgeable about the modes of transmission of STIs and HIV. As in the case of adolescent pregnancy, they do not have a negative attitude towards condoms; however, they consider that there are social norms that challenge women when they take the initiative in using contraceptive and protective methods or ask their partners to do so. Urban adolescents believe that it is time to change this type of norm. In rural areas, negative attitudes persist towards homosexuals and lesbians that reproduce stigmatisation, associating HIV and AIDS as exclusive to this population. As with contraceptive methods, the results in relation to STIs, including HIV/AIDS, are much lower among study participants than those found in the adolescent population at the national level in 2015.^25^

Adolescents of both sexes in urban and rural areas are not knowledgeable about GBV, tending to associate the concept of violence with family arguments or common delinquency. Although GBV is present in most of their daily life environments, they do not identify it as such in principle. They generally only identify sexual aggression and, in a few cases, street harassment as gender-based violence. Positive or condescending attitudes persist towards forms of gender-based violence, such as street harassment or pregnancy of under-age adolescent girls by older men, which are rejected only by urban adolescent girls.

These attitudes all fall under a normative framework which on many occasion normalises gender roles and violence, evident in the conception of the passive posture of women in sexual relations, the mistrust of women who use or ask for contraceptives and/or protection, the moral judgment against the VIP, the tolerance of affective and sexual relations and pregnancies of girls and adolescents with older men. In the case of men, in many cases it is normal to justify violence against women, blaming the victims for provoking it: in the case of physical and psychological mistreatment, for being rude or “answering back”, violating the man’s masculinity, and in the case of sexual harassment or violence, for inciting it by their dress or behaviour. These data also contrast with the 2015 national data in which a less favourable attitude towards gender-based violence was evident among male adolescents at the national level.^25^

Female adolescents recognise themselves as being subordinated to men in situations of courtship, use of contraceptive methods and violence. Since they feel that they have no agency over their bodies and decisions, they assume it is not necessary for them to have knowledge about their rights or about the minimum elements of sexual and reproductive health, especially in rural areas. Of course, this is not a choice; the fact that they do not have access to information or develop reflexive processes about their bodies and decisions is due to hegemonic power structures where the masculine dominates and the feminine complies without any kind of questioning.^30^

These findings, viewing the sexuality of adolescents as a product of multiple determinants,^31,32^ should be situated in a context in which adolescents of both sexes have not had access to or even been offered high-quality CSE^1,3^ or health and protection services, with interdisciplinary and specialised teams that lead them to understand and experience their sexuality in a holistic way and also guarantee their SRHR, a limitation that is even more severe in rural contexts.^33^ In any case, no specific mention was made of their participation in PESCC or SSAAJ activities, which, in accordance with the evaluation of these programmes, could imply either that they did not have coverage in these municipalities or that their implementation did not have an impacting quality.^11,12^

Only in some cases did participants have access to sporadic talks in their educational institutions, and the difference is evident in the case of the group of 14–17-year-olds in Barranquilla, where they have had a more systematic CSE programme. Although mothers are recognised as a reliable source for those who receive guidance, it is more in the sense of prohibitions and judgments than in accompaniment for informed decision making. In fact, in the urban context, on the contrary, adolescents between 10 and 13 years of age consider that sexuality continues to be a taboo for their guardians.

In general, male and female adolescents, both urban and rural, do not know how to access health services and do not have a favourable attitude towards this type of services. They assume that if they ask about sexuality in health services, they will be judged or mocked, and they consider that the health care facilities are in a poor condition. In the case of protection services, there is partial knowledge of the access routes. However, the adolescents do not trust that these are effective. They consider that there is no institutional response to gender-based violence, since neither at home, nor at school, nor in the institutions are they believed when they report any type of violence.

It was evident that the vulnerability resulting from the migrant condition makes the conditions more complex for the Venezuelan adolescent immigrant population. This is not just because of the limitations on access to health services, but also because they are victims of stigmatisation, especially women, including from some protection officials from the rural areas.

According to our findings, some recommendations are established for the *Tírala Plena* project and for interventions on sexuality with adolescents and young people within similar contexts and characteristics.

### Recommendations for the design of a CSE curriculum for *Tírala Plena*

Given the little knowledge that the adolescent population has about their sexuality, the curriculum proposal must start from basic knowledge about sexuality at any age in every context. One of the key aspects to deepening the intervention is gender-based violence, which is reproduced when it is not identified, as there are tolerant attitudes towards it and to a large extent it has been normalised.

As adolescents are not always open to talking about their sexuality, it is necessary to generate contextualised dialogic strategies that allow the building of trust and make it possible to build confidentiality agreements in the group.^27–29^ Likewise, given the lack of trust in parental figures that exists among the adolescent population, the importance of inter-generational training spaces that allow for the development of skills to discuss sexuality in adolescence in these two populations is affirmed.

In the same vein, and in order to strengthen knowledge, trust and access to health and protection services, it is suggested to include not only information on their location, importance and usefulness, but also to invite officials to the sessions, as well as to make guided tours of familiarisation with the health and protection services offices and personnel.

Given the existing differences by gender, specifically related to GBV, it is suggested that during the delivery of comprehensive sexuality education, mixed spaces be combined spaces for men and women differentiated by sex and even by age.

While it is true that there are no significant differences between Venezuelan adolescent migrants and the host population in the studied areas, it is very important to consider language and personal life stories in the provision of CSE, as well as to follow up on the processes of normalisation of the status of Venezuelan adolescent migrants, to guarantee their access not only to education but also to health services, including access to services related to their SRH.

Finally, it is important to highlight the relevance of working outside the school setting from the approaches proposed by the CSE guidelines,^4^ such as the rights approach (sexual and reproductive rights, differential, gender and transformative approach).

## Conclusions

The literature review showed that there is no systematic and updated information on the sexual and reproductive health of adolescents aged 10–17 in the municipalities included in the study. Likewise, the information related to their knowledge, attitudes and social norms regarding sexuality, adolescent pregnancy, STIs including HIV, and gender-based violence is not only partial and outdated but is not disaggregated at the municipal level.

This underlines the relevance of the results of this formative research to developing the pedagogical proposal, the implementation plan and the different components of the CSE strategy.

It was found that the adolescent population of these municipalities have very basic knowledge about their sexuality and particularly about the topics of the study, most of them limited to a biological perspective of sexuality and not about rights.

The attitudes and social norms encountered do not allow for open discussion of sexuality, prevention of teenage pregnancies and gender-based violence; on the contrary, they reproduce gender-based violence. Likewise, there is a lack of quality CSE services for the group of adolescents participating, both in and out of school. The research concludes that the adolescent population in these municipalities has little knowledge and is not accessing SRH services, nor is it using the protection and care routes for GBV cases.

There is a significant presence of Venezuelan adolescent migrants in the areas studied, but there are no major differences between them and the receiving population. Although in some cases these young migrants do not have access to education and health services, some differences appeared only in terms of greater sensitivity of adolescent migrant women from Venezuela to issues related to gender-based violence.

In terms of age, the adolescent population aged 10–13 years showed less knowledge about their sexuality, but reproduced attitudes and social norms like those of older adolescents. In terms of gender, the most important difference is that adolescent women between 14 and 17 years of age have more critical and less normalising attitudes towards sexual violence. In general, in the more rural municipalities, the adolescent population has a greater knowledge of their sexuality and more tolerant attitudes and more permissive social norms regarding GBV.

### Strengths and limitations

The research had some limitations: first, the coverage. The findings would have been richer if a second round of focus groups had been conducted to saturate the categories resulting from the initial findings from the participants. Second, although Venezuelan migrant adolescents participated in the different groups with adolescents from other vulnerable contexts, some specific sessions with them would have allowed us to go deeper into their particular situation. Third, although adolescents generally expressed that they have not had access to quality CSE, we were unable to establish differences in knowledge, attitudes and social norms between schooled and non-schooled adolescents. Finally, the restrictions due to the Covid 19 pandemic led us to conduct some memory workshops and focus groups with adolescents from Barranquilla, as well as the meetings with fathers, mothers, and guardians from Santa Lucía, Barranquilla and Soledad, as focus groups through collective telephone calls. However, not having direct visual contact allowed a kind of anonymity that generated more trust and openness. Thus, participants were able to freely express their points of view and the purposes for which these techniques were designed were achieved.

## Footnotes

* This project is part of a study design led by the World Health Organization (WHO) for multi-country implementation research to assess the feasibility, acceptability and effectiveness of different packages of actions to prepare and support facilitators to deliver CSE in out-of-school settings to defined groups of young people with varying needs and circumstances.

† Colombia's law recognises under-age as below 18 years old. However, the "criminal code" considers sexual abuse any sexual act with 14-year-old children.

[†] In Colombia abortion was considered legal when: (a) the life or health of the mother is in danger, (b) there is a malformation of the fetus incompatible with life and (c) the pregnancy is the result of abuse, rape, incest, egg transfer or non-consensual insemination (Sentence C-355 of 2006). Since Ruling C-055 of 2022, abortion is not a crime before the 24th week of gestation.

## References

[1] IPPF. Derechos sexuales: Una declaración de IPPF Guía de bolsillo; 2009. Available from: https://www.ippf.org/sites/default/files/ippf_sexual_rights_declaration_pocket_guide_spanish.pdf

[2] UNESCO. Educación integral de la sexualidad: Conceptos, enfoques y competencias; 2014. Available from: https://unesdoc.unesco.org/ark:/48223/pf0000232800.

[3] Haberland N, Rogow D. Sexuality education: emerging trends in evidence and practice. J Adolesc Health. 2015;56:S15–S21. doi: 10.1016/j.jadohealth.2014.08.013 25528976

[4] UNFPA, UNESCO, WHO. International Technical and Programmatic Guidance on Out-of-School Comprehensive Sexuality Education (CSE). An evidence-informed approach for non-formal, out-of-school CSE programmes that aims to reach young people from left-behind populations. New York: UNFPA; 2020. Available from: https://www.unfpa.org/publications/international-technical-and-programmatic-guidance-out-school-comprehensive-sexuality 10.1080/26410397.2023.2242175 PMC10431723 37577783

[5] Kim CR, Free C. Recent evaluations of the peer-Led approach in adolescent sexual health education: a systematic review. Perspect Sex Reprod Health. 2008;40:144–151. doi:10.1363/4014408 18803796

[6] UNFPA. State of world population 2019 Unfinished business the pursuit of right and choices for all. Available from: https://www.unfpa.org/sites/default/files/pub-pdf/UNFPA_PUB_2019_EN_State_of_World_Population_0.pdf.

[7] Observatorio de Venezuela y Fundación Konrad Adenauer. Retos y oportunidades de la integración migratoria: análisis y recomendaciones para Barranquilla; 2020. Available from: https://www.kas.de/documents/287914/0/Migracion(BARRANQUILLA-AJ-03-12-2020.pdf/ba201662-6f9d-4cdc-3a1b-e32b2582aeac?t=1607987521705.

[8] Proyecto Migración Venezuela Informe de niñez migrante. Caracterización de la niñez y adolescencia migrante en Colombia. USAID; 2021. Available from: https://migravenezuela.com/informes/caracterizacion-de-la-ninez-y-adolescencia-migrante-en-colombia/

[9] Goldfarb ES, Lieberman LD. Three decades of research: the case for comprehensive sex education. J Adolesc Health. 2021;68:13–27. doi:10.1016/j.jadohealth.2020.07.036 33059958

[10] Makleff S, Garduño J, Zavala RI, et al. Preventing intimate partner violence among young people—a qualitative study examining the role of comprehensive sexuality education. Sex Res Soc Policy. 2020;17:314–325. doi:10.1007/s13178-019-00389-x

[11] Ministerio de Educación Nacional, Universidad de los Andes. Evaluación del Programa de Educación para la Sexualidad y Construcción de Ciudadanía (PESCC). Informe Final; 2014. Available from: https://fys.uniandes.edu.co/site/index.php/component/docman/doc_download/7-informe-evaluacion-programa-de-educacion

[12] Fondo de las Naciones Unidas para la Infancia UNICEF. Consultoría para la evaluación de los Servicios Amigables de Salud para Adolescentes y Jóvenes en Colombia. Producto 5: Informe de Resultados Econometría Consultores; 2014.

[13] Ministerio de Educación Nacional. Circular Conjunta No. 16. Instructivo para la atención de niños, niñas y adolescentes procedentes de Venezuela en los establecimientos educativos colombianos. Bogotá; 2018. Available from: https://www.mineducacion.gov.co/portal/normativa/Circulares/368675:Circular-Conjunta-N-16-de-2018

[14] Ministerio de Educación Nacional. Circular Conjunta No. 16. Instructivo para la atención de niños, niñas y adolescentes procedentes de Venezuela en los establecimientos educativos colombianos. Bogotá; 2018.

[15] Riaño P. Migraciones forzadas y crisis de la memoria: los talleres de memoria con población desplazada en Colombia [documento 17]. Vancouver: UBC; 2004.

[16] Strauss A, Corbin J. Bases de la investigación cualitativa. Técnicas y procedimientos para desarrollar la teoría fundamentada. Colección Contus. Medellín: Universidad de Antioquia; 2002.

[17] [MSPS:]. Ministerio De Salud y Protección Social. SISPRO; 2019 (corte 2018).

[18] Royo M, Forero L, Rivillas J, et al. Evaluación de las necesidades insatisfechas en salud sexual y salud reproductive de la población migrante venezolana en cuatro ciudades de la frontera colombovenezolana. Bogotá: Profamilia; 2019. Available from: https://profamilia.org.co/wp-content/uploads/2019/05/LIBRO-Evaluacion-de-las-necesidades-insatisfechas-SSR-y-Migrantes-Venezolanos-Digital.pdf

[19] INS –Instituto Nacional de Salud –Informe evento VIH/SIDA, 2019.

[20] [Interview] with the Secretary of Public Health of Municipality of Santa Lucía. (2020.

[21] [Base] de Datos Gestantes, Municipio Campo de la Cruz. (2020).

[22] [Interview] with the Secretary of Public Health of Municipality of Barranquilla. (2020).

[23] Guerra R, Aldana E, Rojas I. Conocimiento sobre prevención de infecciones de transmisión sexual en adolescentes inmigrantes habitantes en Soledad-Atlántico 2018-2019. Identidad Bolivariana. 2020; 4(1): 5--15. doi:10.37611/IB4ol15-15

[24] USAID, ONU MUJERES, Universidad del Norte, MINSALUD. Situación de violencias basadas en género de población colombiana y venezolana en Barranquilla; 2019. Available from: https://data.unhcr.org/en/documents/download/73345

[25] Ministerio de Salud y Protección Social y Profamilia. Encuesta Nacional de Demografía y Salud 2015 (tomo II). Bogotá. Ministerio de Salud y Protección; 2016. Available from: https://profamilia.org.co/wp-content/uploads/2019/05/ENDS-2015-TOMO-II.pdf.

[26] Suárez L, Mendivil C, Vega J. jovenHablajoven: una experiencia de comunicación y salud en una población del Caribe colombiano: los y las jóvenes urbano/rurales, su cultura y sus identidades alrededor de la sexualidad. Rev Investig Desarro. 2004;12(1):210–237. Available from: https://rcientificas.uninorte.edu.co/index.php/investigacion/article/view/1099.

[27] Vega, J, Mendivil, CJ. HABLA joven: lessons learned about interpellation, peer communication, and second-generation edutainment in sexuality and gender projects among young people. In: Waisbord, S., Obregón, R. editors. The handbook of global health communication. Oxford: Wiley-Blackwell. 2012. p. 444–468. doi:10.1002/9781118241868.ch21

[28] Beltrán R, Vega J. Aprendizajes sobre la evaluación del diálogo y el debate en estrategias de comunicación y cambio social. El caso de la estrategia de Eduentretenimiento + Movilización Social = Cambio Social. Rev Investig Desarro. 2012;20(2):390–415. Available from: https://rcientificas.uninorte.edu.co/index.php/investigacion/article/view/3771.

[29] Pérez-Llerena Y, Vega-Casanova J. Edu-entretenimiento y movilización social en la prevención de las Violencias Basadas en Género en adolescentes: hallazgos sobre el rol del diálogo, el debate y la reflexión en un piloto de "Revelados" en un municipio del Caribe colombiano. Rev Investig Desarro. 2020;28(1):6–35. doi:10.14482/indes.28.1.323.34

[30] Sánchez M, Sánchez I, Beyer M, et al. Factores asociados al abuso de menores, resultados de una intervención para el fortalecimiento de prácticas de crianza. Rev Puertorriqueña Psicol. 2018;29(1):16–35. Available from: https://www.repsasppr.net/index.php/reps/article/view/399.

[31] Vargas E, Flórez CE, Cortés D, et al. Embarazo temprano. Evidencias de la investigación en Colombia. Bogotá: Ediciones Uniandes; 2019.

[32] Kirby D, Coyle K, Rolleri L, et al. Reducing adolescent sexual risk: A theoretical guide for developing and adapting curriculum-based programs. Scotts Valley: ETR Associates; 2011. Available from: https://www.etr.org/healthsmart/assets/File/resources/ReducingAdolescentSexualRisk.pdf.

[33] Rojas A. Veinte barreras que impiden hablar de sexualidad con niñas, niños y adolescentes; 2020.

